# Dynamic Changes in miRNA Expression during the Generation of Expanded and Activated NK Cells

**DOI:** 10.3390/ijms241713556

**Published:** 2023-08-31

**Authors:** Chantal Reina-Ortiz, Mª Pilar Mozas, David Ovelleiro, Fei Gao, Martín Villalba, Alberto Anel

**Affiliations:** 1 Apoptosis, Immunity and Cancer Group, Department Biochemistry and Molecular and Cell Biology, Aragón Health Research Institute (IIS-Aragón), University of Zaragoza, 50009 Zaragoza, Spain; reinaortiz@msn.com (C.R.-O.); pmozas@unizar.es (M.P.M.); 2 Peripheral Nervous System, Vall d’Hebron Institut de Recerca (VHIR), 08035 Barcelona, Spain; david.ovelleiro@vhir.org; 3 Institute of Regenerative Medicine and Biotherapy, University of Montpellier, INSERM, CNRS, University Hospital Center Montpellier, 34000 Montpellier, France; fei.gao2@inserm.fr (F.G.); martin.villalba@inserm.fr (M.V.); 4 Immuno-Oncology Laboratory, School of Basic Medicine, Central South University, Changsha 410017, China

**Keywords:** NK cells, miRNA, cytotoxicity, immunotherapy

## Abstract

Therapies based on allogenic Natural Killer (NK) cells are becoming increasingly relevant, and our laboratory has produced expanded and activated NK (eNK) cells that are highly cytotoxic against several hematological cancers when used alone or in combination with currently approved therapeutic monoclonal antibodies. In order to produce eNK cells, healthy human donor NK cells undergo a 20-day expansion protocol with IL-2, IL-15 and Epstein–Barr virus (EBV)-transformed lymphoblastoid feeder cells. In order to produce an even more potent eNK-based therapy, we must elucidate the changes our protocol produces within healthy NK cells. To understand the post-transcriptional changes responsible for the increased cytolytic abilities of eNK cells, we performed microRNA (miRNA) expression analysis on purified NK cells from day 0 and day 20 of the protocol using quantitative reverse transcription PCR (RT-qPCR). Of the 384 miRNAs profiled, we observed changes in the expression of 64 miRNAs, with especially significant changes in 7 of them. The up-regulated miRNAs of note were miRs-146a, -124, -34a, and -10a, which are key in the regulation of cell survival through the modulation of pro-apoptotic genes such as *PUMA*. The down-regulation of miRs-199a, -223, and -340 was also detected and is associated with the promotion of NK cell cytotoxicity. We validated our analysis using immunoblot and flow cytometry studies on specific downstream targets of both up- and down-regulated miRNAs such as PUMA and Granzyme B. These results corroborate the functional importance of the described miRNA expression patterns and show the wide variety of changes that occur in eNK cells at day 20.

## 1. Introduction

Properly capitalizing on allogeneic natural killer (NK) cell-based therapies necessitates a better understanding of the transcriptomic changes that occur during the expansion and activation process. NK cells are critical components of the innate immune system that provide an early defense against viral, bacterial, and aberrant tumor cells. The presence of stress ligands on the surface of tumor cells and, on many occasions, their reduction or loss of HLA-I molecules makes them good targets for the action of properly activated NK cells [1,2]. It is worth noting that, due to the inhospitable tumor microenvironment and other immunosuppressive factors, patient NK cells are unable to carry out their cytotoxic function in many instances. In order to re-capture this function, allogeneic NK cells produced from healthy human donors have become increasingly of interest as a cancer treatment, and several clinical trials are underway [3,4].

Pioneering work by the group of Dario Campana developed optimal protocols for activation and expansion of allogeneic or autologous NK cells, initially based on the use of K562 feeder cells transfected with membrane-bound Il-15 and the 41BB ligand [5]. Other groups developed successful expansion protocols based rather in stimulation with a cytokine cocktail containing IL-12, IL-15, and IL-18, generating cytokine-induced killers (CIK), with features of memory-like NK cells [6]. Many other protocols have been developed, using not only cells from adult healthy donors or patient NK cells, but also cells from umbilical cord blood or derived from pluripotent stem cells [7], some of them already in clinical trials [4,8]. We developed a NK cell activation and expansion protocol in which we established an optimal combination of cytokines and feeder cells to produce highly cytotoxic expanded and activated NK (eNK) cells from healthy human donor peripheral blood or from umbilical cord blood [9,10,11]. These expansion protocols would guarantee the use of NK cells from one allogeneic donor to treat one patient in future clinical applications. Over the 20-day expansion period, eNK cells increase expression of CD56 and NKp44 while retaining a constant expression of CD16. In addition, the cytotoxic potential of eNK cells is very much increased compared to freshly isolated NK cells from the same donors, and they exert marked cytotoxicity against cells from a variety of malignancies, especially when combined with therapeutic antibodies directed against tumor antigens such as CD20, EGFR, HER-2 [11], or CD38 [10]. Samples from patients with B-cell chronic lymphocytic leukemia (B-CLL) and multiple myeloma (MM) were treated ex vivo with the eNK cells, alone and in combination with monoclonal antibodies such as daratumumab (anti-CD38) and/or pembrolizumab (anti-PD1) [9,10]. In our MM study, eNK cells were produced from two different sources: umbilical cord blood and healthy adult donor peripheral blood. Each of the eNK cell types exerted a cytotoxic effect against the MM cells in several scenarios. eNK cells from peripheral blood benefited in potency from the addition of daratumumab, thereby suggesting a compelling combination therapy option [10]. Discovering the miRNA changes that produce our eNK cells will present us with previously unknown targets to exploit when creating a more potent cell-based therapy.

MicroRNAs (miRNAs) are small, noncoding RNA molecules that act as regulators of cell development, proliferation, and differentiation [12]. They post-transcriptionally regulate the expression of their target genes, whereby a single miRNA can regulate several genes. miRNAs negatively regulate gene expression by binding to the 3’- untranslated region (3’URTR) of messenger RNAs (mRNAs), which degrades the mRNAs or causes transcript destabilization [13]. Altered miRNA expression, such as overexpression, can promote oncogenes or suppress tumor-suppressing genes [14]. Understanding the impact of miRNA expression changes in healthy cells is equally relevant. 

In previous work, we examined the changes in mRNA and miRNA expression in NK cells activated in 5-day protocols with IL2 alone, with IL2+K562 cells, or with EBV^+^ R69 cells. These protocols did not result in prominent NK cell expansion, although they increased the cytotoxic potential of the NK cells [15]. miRNA expression analysis resulted in the identification of miR-23a as a key regulator of active granzyme B expression in activated NK cells. In this study, we went beyond the previous 5-day protocol and expanded and activated NK cells for a full 20 days. Using RT-qPCR, we examined the miRNA profiles of day 0 and day 20 NK cells. We then validated our findings using Western blot and flow cytometry studies. We sought to reveal the transcriptomic changes that occurred after the 20-day protocol was completed that allowed for eNK cells to have a greater cytolytic advantage over resting NK cells in NK-cell-based therapies.

## 2. Results

### 2.1. Expression of miRNAs in Healthy Donor NKs at Day 0 and Day 20 of Expansion 

**Figure 1:**
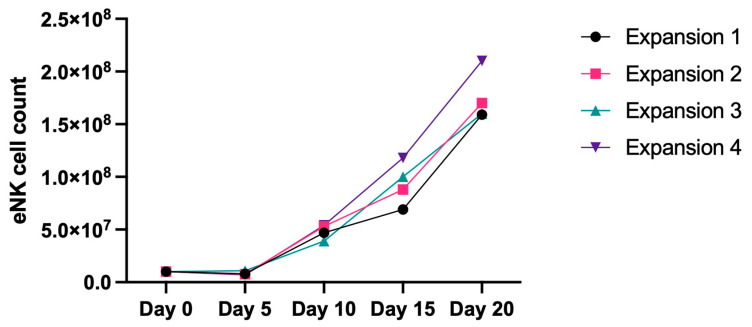
Total number of CD56^+^ NK cells during the expansion protocols. The graph indicates the total number of NK cells at each stage of the expansion and activation protocol. The four expansions used for transcriptomic analysis are shown. Total number of NK cells is based on the number of CD56^+^CD3^−^ cells in each culture as verified via flow cytometry staining. Greater than 90% eNK cell purity was reached by day 20 on all expansions.

To establish the miRNA signatures of both resting and expanded NK cells, we purified NK cells from the peripheral blood of healthy human donors. Samples were taken at day 0 and day 20 once the expansion protocol was successfully completed. We tested four matched sets of eNK cells totaling eight samples. The increase in NK cell numbers over the course of these expansions is shown in Figure 1. The mean expansion of NK cells in these four donors was 162-fold, with a maximum expansion of 200-fold in expansion #4, as expected using our protocol (see refs. [9,11]). Each expansion would be sufficient to treat a single patient. 

**Figure 2:**
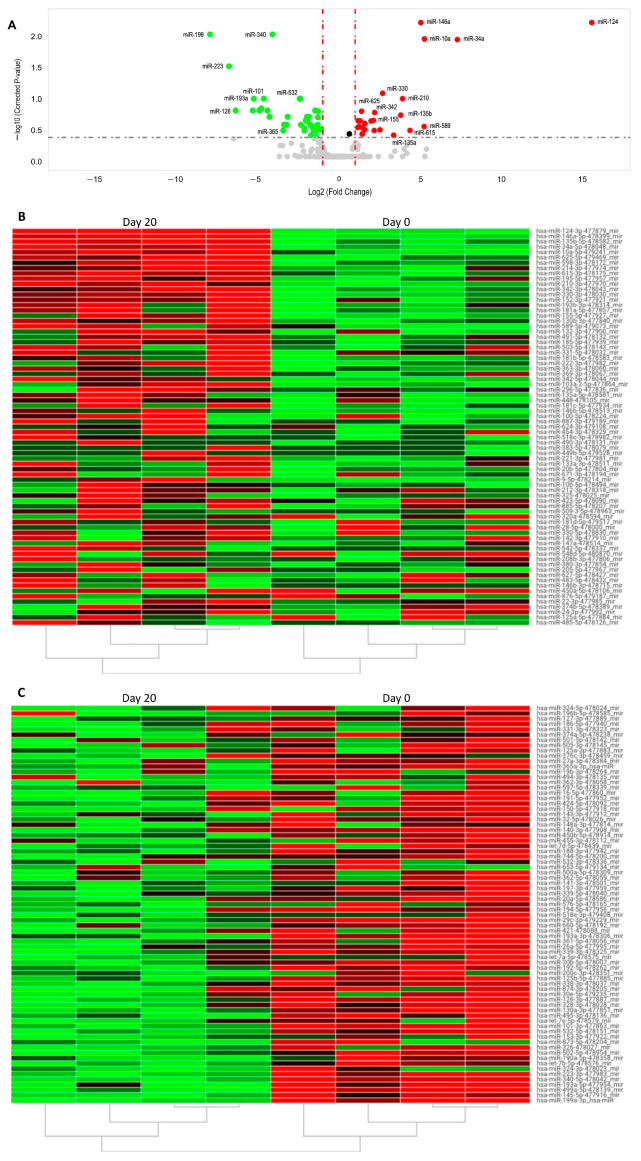
miRNA expression profile of eNK cells. (**A**) Volcano plot presenting the sum of eNK miRNAs differentially expressed between day 0 and day 20 of expansion. miRNA microarray ex-pression profiling from 4 paired NK cell expansions was performed. The threshold to identify up and down regulated genes was a fold change ≥ 2 and −log10 (corrected *p*-value) < 0.1. Red dots in-dicate differentially up-regulated miRNAs and green dots indicate differentially down-regulated miRNAs. The black dot signifies a miRNA with null change while the grey dots indicates miRNAs with insignificant differential expression. (**B**,**C**) Heat maps showing hierarchical clustering of differentially expressed miRNAs from day 20 compared to day 0. Each column represents one NK sample (either from day 20 or day 0) and each row represents one miRNA. Relative expression is indicated using a color scale. Red tones are indicative of up-regulation (−1.1) and green tones are indicative of down-regulation (1). Full hierarchical clustering can be found as Figure S1 in Supplemental Figures.

Then, using the TaqMan microRNA assay, we determined the changes in miRNA expression levels prompted by our expansion protocol in purified NK cells at day 0 and at day 20 of the expansions. We chose this assay because it contained all the relevant miRNAs that we wanted to explore. The day 0 samples and day 20 samples were analyzed individually, paired, then compared as two groups using ThermoFisher analysis software version 4.3 (v4.3). The ΔΔCTs were calculated using the day 0 values as a reference. All CT values above 35 were not considered valid as too many PCR cycles were required for detection, indicating low expression levels (Supplementary Figure S1). Of the 384 miRNAs quantified (see Supplementary Figure S2), 64 were considered differentially expressed: 39 were down-regulated and 25 were up-regulated, represented as a volcano plot in Figure 2A. From these data, we calculated the *p* values with the Applied Biosystems qPCR analysis application based on comparing the ΔΔCt values using multivariate analysis, considering statistically significant *p* values ≤ 0.05. After this analysis, the statistically significant down-regulated miRNAs were: miR-199a, miR-223, and miR-340 (Table 1). The four significantly up-regulated miRNAs were: miR-124, miR-10a, miR-34a, and miR-146a (Table 1). Figure 2B,C show the expression pattern in each of the samples using heat-maps corresponding to the up- and down-regulated miRNAs, respectively. 

### 2.2. Down-Regulated Differentially Expressed miRNAs

The miRNA with greatest down-regulation at day 20 when compared to expression levels at day 0 was mir-199a. By day 20, mir-199a expression was down-regulated with a fold change (FC) of 0.004 (*p* < 0.01), a statistically significant reduction. This miRNA has been studied in relation to different neoplastic and neurodegenerative diseases, particularly hepatocellular carcinoma [16,17,18,19,20,21]. The expression of miR-199a is down-regulated in hepatocellular carcinoma (HCC) compared with healthy donor samples, and this has been associated with the regulation of the pro-apoptotic gene *PUMA* [22]. Low levels of mir-199a in HCC cells correspond to low levels of PUMA, resulting in an anti-apoptotic effect, prolonging the survival of cancer cells. As such, mir-199a has become a therapeutic and diagnostic target in HCC and many other malignant tumors. In our study, mir-199a was also down-regulated at day 20, indicating that eNK cells can undergo changes in apoptotic pathways in order to prevent cell death. 

**Figure 3:**
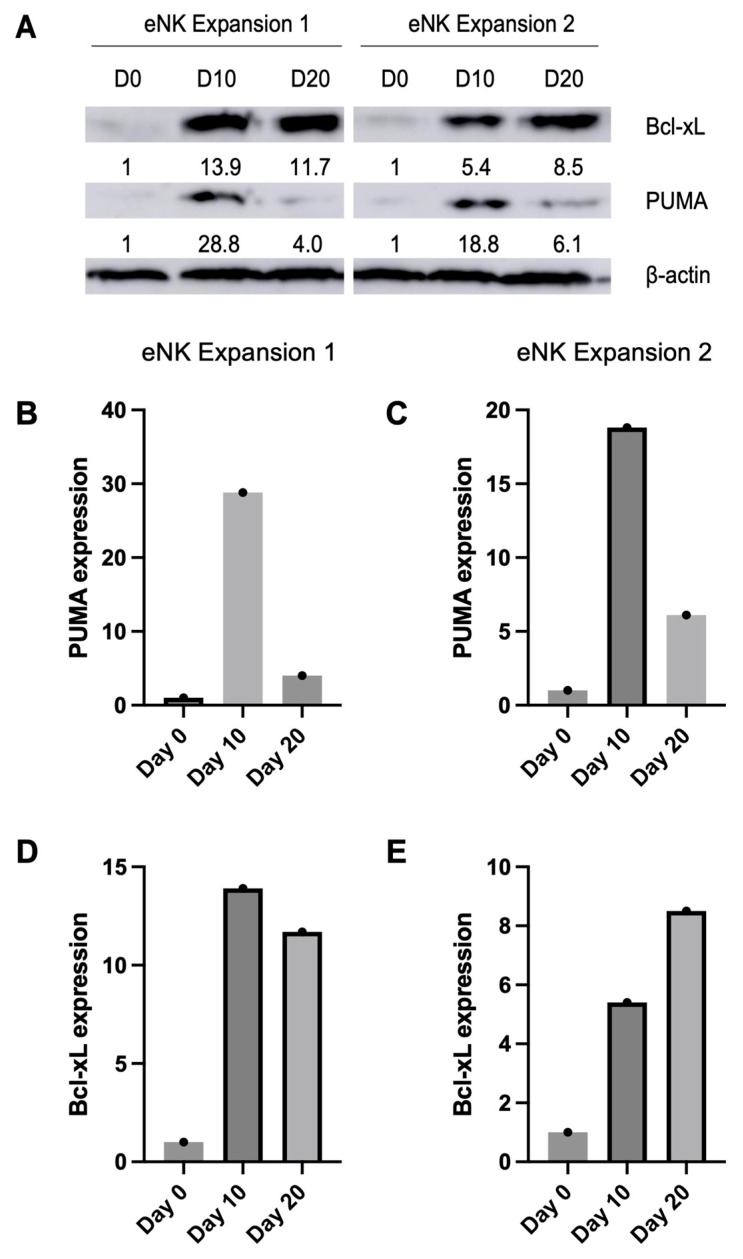
Validation of PUMA and Bcl-x_L_ expression levels using Western blot based on miR-199a findings. (**A**) Expression levels of Bcl-x_L_ and PUMA were determined via Western blotting in expansions 1 and 2. Samples were taken at day 0, 10, and 20 of the expansion. (**B**,**C**) Bar graphs show the quantified expression of PUMA at each stage of the expansion for eNK expansion 1 (**B**) and 2 (**C**). (**D**,**E**) Bar graphs showing the quantified expression of Bcl-x_L_ at each stage of the expansion for eNK expansion 1 (**D**) and 2 (**E**). Day 0 is used as point of reference and protein control was β-actin. Intensities of the bands were determined densitometrically. Protein samples obtained from two independent samples in each expansion were subjected to Western blot analysis, and results are shown as mean ± SD.

The progression of PUMA expression was analyzed in eNK samples taken at D0, D10, and D20 of expansion and activation (Figure 3A). The PUMA levels at day 0 were extremely low and used as the point of comparison. Day 10 presented the highest levels of PUMA in the samples analyzed, with a mean expression of 23.8. However, by day 20, we observed a marked reduction in PUMA expression, averaging 5.05; a 79% decrease from day 10. Graphs detailing each expansion’s PUMA progression can be found in Figure 3B,C. In the case of miR-222, there was a greater than two-fold increase at day 20, although not statistically significant. While mir-222 has not been directly studied in NK cells, it has been studied in a variety of cells, particularly in various cancers [23,24,25]. In these studies, miR-222 has been established as a powerful negative regulator of PUMA. The expression of PUMA is suppressed by miR-222, preventing the induction of cellular apoptosis [21,26]. The down-regulation of miR-199a and the up-regulation of miR-222 contribute to reduce PUMA expression levels at day 20, having an anti-apoptotic and pro-survival effect on eNK cells.

Although not statistically significant, miR-326, miR-140, and let-7b were also down-regulated, presenting FC values of 3.88, 3.29, and 3.61, respectively. These miRNAs have been shown to directly regulate Bcl-x_L_, an anti-apoptotic protein from the Bcl-2 family, in a variety of cell types. miR-326 and let-7b have been shown to inhibit Bcl-x_L_ expression in stored platelets [27,28]. In vascular smooth muscle cells, the increased expression of miR-140 induced a down-regulation of Bcl-x_L_ [29]. In hepatocellular carcinoma, let-7b also negatively regulates Bcl-x_L_ expression, potentiating the effect of sorafenib-induced apoptosis by curtailing the anti-apoptotic function of Bcl-x_L_ [30]. In our eNK cells, we examined Bcl-x_L_ expression alongside PUMA using immunoblot analysis (Figure 3A). The day 0 expression levels were very low and were used as the point of comparison. Both of the eNK cell expansions analyzed had increased levels of Bcl-x_L_ at day 10 when compared to day 0; expansion 1 had its peak expression at day 10 with a slight decrease at day 20 (Figure 3D, while expansion 2 had its peak Bcl-x_L_ expression at day 20 (Figure 3E). Altogether, the eNK cell expansions maintained elevated expression of Bcl-x_L_ throughout the expansion when compared to day 0. 

**Figure 4:**
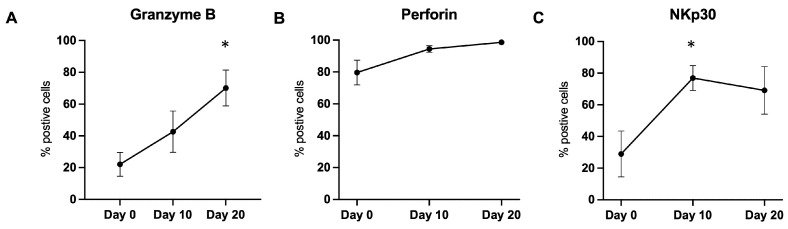
Flow cytometry analysis of granzyme B, perforin, and NKp30 expression in NK cells throughout the 20-day activation and expansion protocol. Levels of each were studied at 10-day intervals. (**A**) Granzyme B expression was analyzed intracellularly with a significant increase at day 20 as compared to day 0. (**B**) Perforin levels were studied intracellularly and remained steady throughout the 20 days. (**C**) NKp30 was studied extracellularly and had significant peak expression as day 10 as compared to day 0. * *p* ≤ 0.05.

Our second most down-regulated miRNA was miR-223-3p (fc = 0.009; *p* < 0.05 *). This miRNA has been identified as being down-regulated in NK cells in a time-dependent manner upon exposure to IL-15 [31]. Granzyme B has also been identified as its direct target in NK cells. At rest, baseline miR-223 expression prevents granzyme B production. However, upon activation through the use of IL-15, miR-223 is down-regulated and granzyme B expression is increased. We observe this same pattern of expression in eNK cells with a steady increase in granzyme B being produced over the course of 20 days, with the highest levels recorded on day 20 (Figure 4A). Granzyme B is also regulated by miR-27a [15]. As part of the miR-23a~27a~24-2 locus, miR-27a negatively regulates both granzyme B and perforin expression in NK cells [32]. Our analysis showed a greater than 80% down-regulation of miR-27a, which, while not statistically significant, did correlate to the increase of both granzyme B and perforin in eNK cells over the 20-day expansion (Figure 4A,B).

The third miRNA that was significantly down-regulated was miR-340-5p (fc = 0.058, *p* < 0.01). No studies are available on the role of miR-340-5p in NK cells. The down-regulation of miR-340 promotes the migration, cell proliferation, drug resistance, and invasion of several types of cancer cells, including colorectal and squamous cell carcinoma [33,34]. The possible downstream targets include ANXA3, PERK, and PNO1, all of which are negatively regulated [35,36]. Regarding NK cells, these properties can be translated to increased migration to target tissues, cell proliferation, and resistance to apoptosis. 

### 2.3. Up-Regulated Differentially Expressed miRNAs

Of the 384 miRNAs analyzed, 4 were significantly up-regulated: miR-124, miR-34a, miR-10a, and miR-146a. 

The role of miR-124 has not been directly studied in NK cells. However, miR-124 directly regulates Signal Transducer and Activator of Transcription 3 (STAT3) in several cell types [37]. STAT3 is constitutively activated in many cancers and its inhibition is being explored in clinical trials [38,39,40]. On the other hand, the loss of STAT3 in murine NK cells resulted in increased expression of granzyme B, perforin, and DNAM-1 [41]. Hence, the increase in miR-124 could be associated with this effect, correlating also with the effect of down-regulation of miR-233 and miR-27a. We described their effects in the previous section and could also correlate with the observed increases in cytotoxicity and in perforin and granzyme B expression (Figure 4A,B). 

NKG2D and NKp30 expression has also been linked to STAT3, with conflicting reports [42,43]. We previously compared the expression of the activating receptors NKG2D and NKp30 at the beginning and end of our activation protocol [9]. We also explored the changes in NKp30 expression in our present study. NKG2D was highly expressed at day 0 and maintained expression throughout the expansion process [9]. NKp30 expression increased significantly with peak expression at day 10, with an average of 78% eNK cells expressing the receptor, a level that was maintained at day 20 (Figure 4C). Thus, the up-regulation of miR-124 can be taken as a negative regulator of STAT3, inhibiting the impact of STAT3 on the expression of NKp30. 

Known as a tumor suppressor and regulator of apoptosis, miR-34a is a transcriptional target of p53 [44]. While miR-34a has not been directly linked to NK cell function, it was significantly up-regulated in eNK cells at day 20 (fc = 159.077; *p* < 0.01). This miRNA is down-regulated in several types of tumor cells when compared to healthy cells [45,46,47]. In cervical cancer, among others, the overexpression of miR-34a results in an increase in cell death and decrease in proliferation due to cell cycle arrest [48]. As evidenced by previously published expansion data and shown in Figure 1, our eNK cells proliferated over 200-fold by day 20; thus, this negative effect on cell growth was not observed in non-tumoral eNK cells.

miR-10a is significantly up-regulated at day 20 (fc = 38.856; *p* < 0.01). The up-regulation of miR-10a has been described in several cancer types [49,50]. In acute myeloid leukemia, miR-10a directly binds to several key p53-dependent genes, suppressing p53 central role in cell cycle arrest and apoptosis [51]. The high levels of miR-10a expressed in eNK cells may have a role in eNK cell proliferation and further strengthen the antiapoptotic changes described in the previous section. As far as we know, this is the first reported analysis of miR-34a and miR-10a expression in activated NK cells. 

Our eNK cells have a unique phenotype and miRNA profile compared to those of standard, non-activated NK cells. This is clear when analyzing the pattern of miR-146a expression, significantly up-regulated at day 20 (fc = 33.218; *p* < 0.0.1). miR-146a has been described as a negative regulator of IFN-γ production in NK cells and is one of the miRNAs most relevant to NK cell function [31]. In agreement with that, our eNK cells show high cytotoxicity but low cytokine production [9].

**Figure 5:**
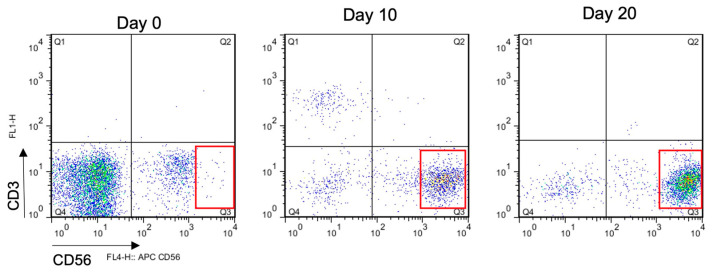
Phenotype progression of NK cells during expansion and activation. Representative dot plots from flow cytometry with X-axis showing CD56 expression and Y-axis showing CD3 expression. Red squares indicate the CD56^bright^ NK cell population that expands and proliferates preferentially during the expansion protocol.

Previous studies focused on NK cells from diseased patients or resting CD56^dim^CD16^+^ cells [52,53]. When miR-146a expression was examined in various NK cell subsets, it was found that CD56^bright^CD16^−^ NK cells expressed high levels of miR-146a with corresponding high levels of IFN-γ after stimulation with IL-12 and IL-18 [54]. However, our eNK cells are CD56^bright^CD16^+^ effector cells [9], with a high capability to exert antibody-dependent cellular cytotoxicity (ADCC) [10,11]. As also shown in Figure 5, there is a progression of CD56 expression from dim to bright during the generation of eNK cells. However, the CD56^bright^ cells that are attained at day 20 are not the same as the regulatory CD56^bright^ cells found in the resting state in lymph nodes. In fact, eNK cells maintain high levels of CD16 throughout the expansion process, as opposed to CD56^bright^CD16^−^ NK cells [10,11]. 

**Figure 6:**
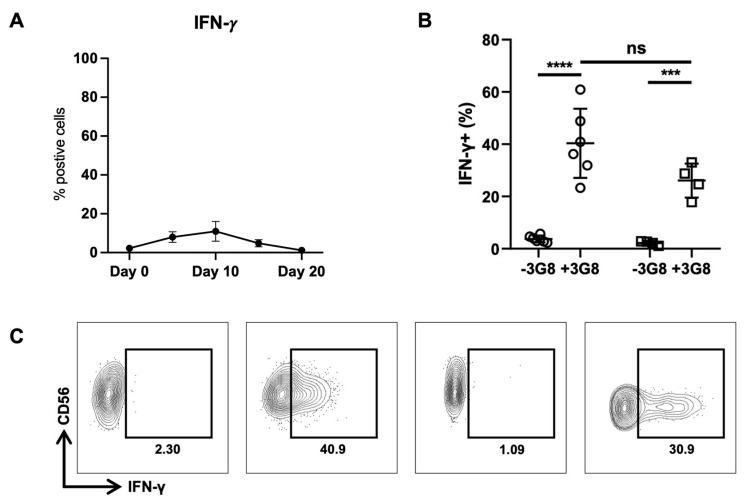
IFN-γ expression in eNK cells. (**A**) Flow cytometry analysis of intracellular IFN-γ during the 20-day expansion protocol. Expression was measured in NK cells every 10 days. (**B**) The percent of IFN-γ production in primary NK cells (left) and eNK cells (right) treated or not with the anti-CD16 agonist mAb 3G8. (**C**) Representative flow cytometry contour plot of IFN−γ secretion in primary (left panels) and eNK cells (right panels) treated (panels 2 &,4) or not (panels 1 & 3) with 3G8. **** *p* ≤ 0.0001, *** *p* ≤ 0.001.

To examine the downstream effects of miR-146a up-regulation, we quantified IFN-γ intracellularly in our eNK cells throughout the 20-day expansion. The percentage of NK cells positive for IFN-γ expression remained very low throughout with peak expression at day 10 that never surpassed 20% of the NK cell population (Figure 6A). In addition, we explored the effect of CD16 ligation by the 3G8 mAb on IFN-γ expression. We confirmed that the basal level of IFN-γ expression in primary NK cells or in day 20 eNK cells is null (Figure 6B,C). When stimulated with 3G8, IFN-γ production levels increased significantly and averaged 40.9% in primary NK cells; meanwhile, although increasing, these levels reached a lower level in eNK cells (only 30.9%). All these results confirm that miR-146a negatively regulates IFN-γ expression in eNK cells. These results confirm the unique changes that occur at both the transcriptomic and protein levels in order to produce effective eNK cells.

## 3. Discussion

In this study, we report changes in the NK cell miRNA profile after expansion. Our expansion protocol is optimized for producing NK cells with high natural cytotoxicity and ADCC against multiple tumor targets in vitro and in vivo [9,10,11,55,56]. Of the 378 miRNAs in the array, we identified 3 miRs significantly down-regulated and 4 significantly up-regulated at day 20, while a total of 64 miRNAs showed some fold variation. Remarkably, the miRNAs significantly affected by the expansion protocol played roles in cell proliferation, apoptosis, and NK cell function. Moreover, we found strong consistency in the miRNAs that increased and decreased, their biological role, and the NK cell physiology. In fact, it is tempting to hypothesize that all these miRNAs are regulated in a “synchronous” manner after NK cells encounter target cells, leading to proper proliferation/expansion and activation. 

A distinct pattern emerged amongst the up- and down-regulated miRNAs. The down-modulated miRNAs are mainly regulated by cytokines, particularly IL-2 and IL-15. Specifically, IL-15 is associated with the down-regulation of mir-223-3p and of the locus miR-23a~27a~24-2, resulting in the increased expression of perforin and granzyme B [15,31,32]. This type of miRNA-dependent gene regulation correlates with the effective increase in cytotoxicity observed in eNK cells, the most important functional feature of these cells for their use as an anti-tumoral treatment [9,10,11]. In relation to the miR-27a locus, our previous studies in 5-day activated NK cells have pointed to the importance of mir-23a in the generation of active granzyme B by the regulation of cathepsin C expression [15]. However, we did not detect the down-regulation of mir-23a in day 20 eNK cells. This could indicate that by day 20, the relevance of mir-23a on granzyme B production waned and there was a change in emphasis towards regulation by miR-27a and miR-223-3p during the prolonged expansion. 

This brings us to a large caveat in comparing our findings to previous studies in which the durations of activation are different [15]. In most of the studies performed, NK cells were activated for 1.5 to 5 days [15,57,58]. Therefore, it is difficult to compare relatively short activation protocols with the generation of cells that have largely proliferated and show high cytotoxic activity. As seen in our immunoblot and flow cytometry data, day 10 is a point of inflection, where the expression of activating receptors and cytokine production take off, and day 20 marks peak viability. Further studies of the transcriptomic changes that occur between day 0 and day 20 are necessary to fully understand the molecular mechanism(s) activated during expansion. However, it is clear that dramatic changes occur at day 10, allowing for the production of robust eNK cells at day 20.

**Figure 7:**
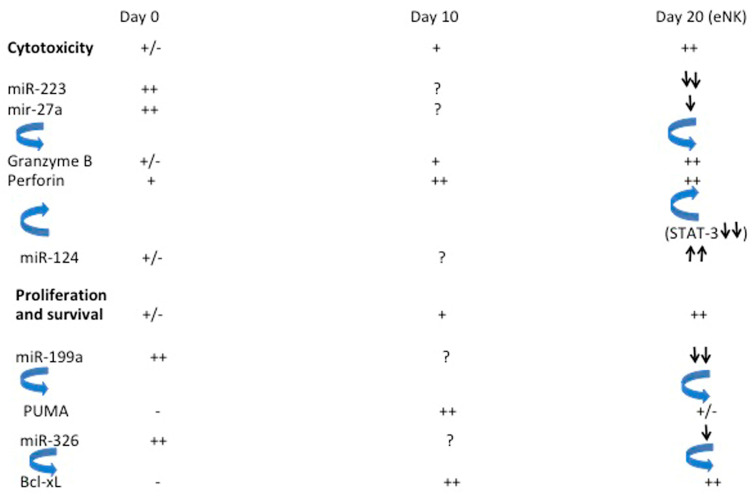
Simplified scheme of the changes in relevant genes and their regulation by specific changes in miRNA expression.

Most studies related to gene regulation by down- or up-regulated miRNAs were performed in cancer cells [33,36,37,50]. We found that eNK cells use similar mechanisms as cancer cells to proliferate and evade apoptosis. The regulation of the pro-apoptotic member of the Bcl-2 family *PUMA* is especially interesting. The expression of this gene is inhibited by mir-199a down-regulation and by miR-222 up-regulation, both being events observed in day 20 eNK cells as compared with day 0 freshly isolated NK cells. While previous reports have shown that NK cells have higher cytotoxic ability beginning at day 5 of stimulation, the exponential expansion of eNK cells does not occur until day 10 [9,10,11,59,60]. This is also the peak of PUMA expression. It seems that, from day 10 on, there is a miRNA-regulated inhibition of PUMA expression, allowing for subsequent exponential growth until day 20. As previously discussed, the concerted action of several miRNAs is required to achieve full NK cell expansion. Additionally, the expression of Bcl-x_L_ remained high at day 20 in comparison to day 0 levels, indicating that eNK cells, once activated, express high levels of Bcl-x_L_ and maintain elevated levels throughout their expansion. Multiple miRNAs contributed to this sustained anti-apoptotic protein expression, allowing for the prolonged life of the eNK cells. The increase in PUMA expression at day 10 was not enough to overcome the anti-apoptotic expression of Bcl-x_L_, as seen by the continued eNK cell expansion after day 10. A simplified schematic showing the effects of these miRNA changes is shown in Figure 7.

## 4. Materials and Methods

### 4.1. NK Cell Activation and Expansion Protocol

As previously described [9,10] healthy human donor leukopacks were obtained from the Blood and Tissue Bank of Aragon, Spain. Peripheral Blood Mononuclear Cells (PBMCs) were isolated using density gradient centrifugation with Histopaque-1077, density 1.077 g/mL (Sigma Life Sciences, Madrid, Spain). The CD3^+^ cell fraction of PBMCs was partially depleted using the EasySep Human CD3 Positive Selection Kit II (Stemcell Technologies, Grenoble, France). The remaining cells, including the CD56^+^CD3^−^ NK cell fraction, were cultured alongside 25 IU/mL of IL-15 and 100 IU/mL of IL-2. Then, 721.221 cells, an EBV+ lymphoblastoid B cell line, were inactivated and used as feeder cells at a ratio of 1:5 with the total number of cells in culture. The combination of these concentrations of IL-15 and IL-2 were used based on our previous studies in which the addition of IFN-**α** was also tested, but did not offer clear advantages over the combination of IL-15 and IL-2 [9]. IL-15 is a cytokine needed to maintain the viability of activated NK cells [61] and IL-2 is needed to guarantee NK cell proliferation, together with the presence of feeder cells [62]. As 721.221 cells are HLA-null, this abrogates inhibitory KIR signaling, lowering the threshold for NK cell activation. The cytokines and feeder cells were refreshed every 5 days of the 20-day protocol. Samples of NK cells were taken at day 0, 10, and 20 of expansion for further study. The NK cell expansion and purity were monitored through flow cytometry every 5 days. The four expansions reached greater than 90% NK cell purity at day 20 as judged by CD56^+^CD3^−^ staining, similarly to results previously published using this protocol [10]. In any case, NK cells were purified before the miRNA determinations via positive selection using anti-CD56 mAb-coated beads from Milteny and magnetic separation. 

### 4.2. Total RNA Extraction 

The total RNA, including the miRNA fraction, was isolated from 8 NK samples (4 paired samples at day 0 and day 20) using the mirVANATM miRNA isolation kit (Thermo Fisher Scientific, Madrid, Spain) and performed according to manufacturer’s protocol. We harvested 10^6^ cells from day 0 and day 20 of each expansion and preserved them using RNA later (Thermo Fisher Scientific, Madrid, Spain) to preserve RNA integrity. The small RNA fraction was not enriched in order to better quantify the amounts of miRNAs in the total RNA fraction. The extracted RNA was quantified using the Qubit Fluorometer (Thermo Fisher Scientific, Madrid, Spain). 

### 4.3. RT-qPCR and miRNA Quantitative Analysis 

The TaqMan™ Advanced miRNA Assay (Thermo Fisher Scientific, Madrid, Spain) was the kit used for RT-PCR. We began by preparing cDNA templates for each sample using the TaqMan™ Advanced miRNA cDNA Synthesis Kit (Thermo Fisher Scientific, Madrid, Spain). We used 2uL of total RNA for each sample. Once the mature miRNAs were reverse transcribed to cDNA, the Universal miR-Amp Primers were added to amplify the amount of cDNA for each target. Once amplified, RT-PCR was performed. The amplified cDNA template was mixed with TaqMan^®^ Fast Advanced Master Mix. A final volume of 10 uL was loaded into the ports of each 384-well Taq-Man^®^ Advanced miRNA Human A card (Thermo Fisher Scientific, Madrid, Spain). The control wells present on each card contained hsa-miR-16-5p, cel-miR-39-3p, and ath-miR159a. RT-qPCR was performed on a ViiATM 7 Real-Time PCR System (Thermo Fisher Scientific, Madrid, Spain). The polymerase activation was performed at 95 °C for 20 s. This was followed by 40 cycles of PCR consisting of denaturing at 95 °C for 1 s then annealing/extending at 60 °C for 20 s. The data were analyzed and the relative expression of each miRNA was calculated using the 2^−ΔΔCt^ method from the Applied Biosystems™ qPCR analysis application (Thermo Fisher Scientific, Madrid, Spain). A Ct (cycle threshold) of 35 was chosen as a cutoff for analyzing samples, as 35 was the cycle before the background signal from control wells began to be amplified (Ct 37+). Wells containing samples with Ct higher than 35 were considered as not expressing the analyzed miRNA and thus not used in our study. The *p*-values shown for RT-qPCR results were derived from the Applied Biosystems qPCR analysis application based on comparing the ΔΔCt values using multivariate analysis. *p* values ≤ 0.05 were considered to be statistically significant. All materials and software used for RT-PCR were sourced from Thermo Fisher Scientific. 

### 4.4. Immunoblot

The NK cells were lysed in lysis buffer (1% Triton-X-100; 150 mM NaCl 50mM Tris/HCl ph7.6; 10% *v*/*v* glycerol; 1 mM EDTA; 1mM sodium orthovanadate; 10 mM sodium pyrophosphate; 10 µg/mL leupeptin; 10 mM sodium fluoride; 1 mM methyl sulfide, Sigma) on ice for 30 min and ultra-centrifugated for 20 min at 12,000 rpm at 4 °C. The protein concentrations were calculated using a BCA assay (Thermo Fisher Scientific). For each sample, 10 µg of protein was loaded and mixed with 3x lysis buffer (SDS 3% *v*/*v*; 150 mM Tris/HCl; 0.3 mM sodium molybdate; 30% *v*/*v* glycerol; 30 mM sodium pyrophosphate; 30 mM sodium fluoride; 0.06% p/v bromophenol blue; 30% *v*/*v* 2-mercaptoethanol, purchased from Sigma). For SDS-PAGE protein separation, a 12% polyacrylamide gel was used. Transfer was performed onto nitrocellulose membranes using a semi-dry electro-transfer method (GE Healthcare, Madrid, Spain). The membranes were subsequently blocked using TBS-T buffer (10 mM Tris/HCl, pH 8.0; 120 mM NaCl; 0.1% Tween-20, 0.1 g/L thimerosal, Sigma, Madrid, Spain) with 5% skimmed milk. Anti-PUMA (SR42-09, Novus Biologicals, Móstoles, Spain) and anti-Bcl-x_L_ (54H6, Cell Signaling, Barcelona, Spain) mAbs were used for Western blot and were incubated overnight in agitation at 4 °C. Appropriate anti-rabbit or anti-mouse peroxidase labeled secondary antibodies (Sigma) were incubated for 1 h at RT. The antibody dilutions used were specified by the manufacturer. The blots were analyzed with Pierce EL Western Blotting Substrate (Thermo Fisher Scientific, Madrid, Spain) using Amersham Imager 680 (GE Healthcare Life Sciences, Madrid, Spain). β-actin expression was used as a reference to normalize the data (Cell Signaling, Barcelona, Spain; Ref. 3700). ImageJ software (NIH, Bethesda, MD, USA) was used to quantify protein expression through densitometry. 

### 4.5. Flow Cytometry 

The intracellular and extracellular staining of NK cells was performed throughout the expansion. We washed 2.5 ×105 cells with PBS and stained them with the appropriate combination of fluorochrome-conjugated monoclonal antibodies for 30 min in the dark. The following antibodies were used in this study: CD56–APC, CD3–FITC (Miltenyi Biotec, Pozuelo de Alarcón, Spain); NKp30 (CD337)–PE (BD Biosciences, Madrid, Spain). Human NK cells are defined as being positive for CD56 staining and negative for CD3 expression, while NKp30 is a characteristic NK-cell-activating receptor. For intracellular staining, 5 × 10^5^ cells were harvested and washed in ice-cold PBS, then fixed in 4% formaldehyde for 20 min at RT. The cells were then permeabilized using 0.1% saponin for 20 min at RT. After fixation/permeabilization, the cells were stained for perforin – REAfinity FITC, IFN-γ–REAfinity FITC (Miltenyi Biotec), and granzyme B–FITC (BD Biosciences). Appropriate isotype-matched controls were included. All stained cells were measured using a FACSCalibur flow cytometer (BD Biosciences), and the data were analyzed using FlowJoTM v7.0 Software (BD Life Sciences, Ashland, OR, USA). To assess NK IFN-γ production using immobilized anti-CD16 Ab 3G8, Nunc™ Immuno Maxisorp 96-Well Microplates were used to coat anti-CD16 agonist 3G8 and the unstimulated group was replaced with 1 × PBS. After overnight incubation at 4 °C, 0.2 million PBMCs or eNK cells were added to each well, along with Brefeldin A, Monensin, and anti-IFN-γ fluorescent conjugated antibodies. The cells were cultured for 6 h in an incubator at 37 °C and 5% CO_2_ before being harvested for flow cytometry. For these stainings, the gating strategy was first the selection of living cells by FSC/SSC, and then the selection of double positive cells for CD56 and for the specific marker in each case. 

### 4.6. Statistical Analysis

Statistical analysis using Student’s *t*-test was performed using GraphPad Prism v9 (GraphPad Software Inc., Boston, MA, USA) unless otherwise noted. *p* values ≤ 0.05 were considered significant. 

## 5. Conclusions

We recently reviewed the current status of NK cell-based immunotherapies in clinical trials as they are of great interest as an off-the-shelf, non-GVHD inducing cellular therapy; several autologous and allogenic NK cell products are currently in clinical trials [4]. As there are several approaches to producing NK cells for clinical use, it is important to identify the transcriptomic changes associated with optimal anti-tumor activity. Identifying key miRNAs and their downstream targets will help in the production of more efficient anti-tumoral eNK cells. Establishing an eNK miRNA-based identity will also help differentiate the treatment of eNK cells from existing patient NK cells. In the present study, we demonstrated that the down-regulation of mir-223-3p and the locus miR-23a~27a~24-2 resulted in the increased expression of perforin and granzyme B, correlating with the effective increase in cytotoxicity observed in eNK cells. On the other hand, the expression of the pro-apoptotic protein PUMA is inhibited by mir-199a down-regulation and by miR-222 up-regulation in eNK cells. Together with the increase in Bcl-x_L_ expression, these changes favor the survival of the highly cytotoxic eNK cells generated. 

## Figures and Tables

**Table 1:** Differentially expressed miRNAs at day 20. The first 3 columns contain the most differentially down-regulated miRNAs at day 20. The last three columns contain the most differentially up-regulated miRNAs at day 20. Listed alongside each target miRNA are their fold change value and *p*-value when compared to day 0 values. miRNAs that were significantly differentially expressed at day 20 are marked with ** and * indicating *p*-values < 0.01 and 0.05, respectively.

Differentially Expressed miRNAs					
Down-Regulated miRNAs			Up-Regulated miRNAs		
Target	Fold Change	*p*-Value, C	Target	Fold Change	*p*-Value, C
hsa-miR-199a-3p	0.004	** 0.01	hsa-miR-124-3p	50,135.445	** 0.01
hsa-miR-223-3p	0.009	* 0.04	hsa-miR-34a-5p	159.077	** 0.01
hsa-miR-126-3p	0.012	0.19	hsa-miR-10a-5p	38.856	** 0.01
hsa-miR-495-3p	0.025	0.19	hsa-miR-589-5p	38.322	0.33
hsa-miR-193a-3p	0.026	0.12	hsa-miR-146a-5p	33.218	** 0.01
hsa-miR-145-5p	0.033	0.19	hsa-miR-615-3p	20.92	0.39
hsa-miR-193a-5p	0.036	0.17	hsa-miR-210-3p	15.214	0.12
hsa-miR-101-3p	0.04	0.12	hsa-miR-135b-5p	14.119	0.22
hsa-miR-130a-3p	0.047	0.19	hsa-miR-135a-5p	10.394	0.46
hsa-miR-338-3p	0.051	0.23	hsa-miR-330-3p	6.473	0.1
hsa-miR-340-5p	0.058	** 0.01	hsa-miR-342-5p	5.791	0.38
hsa-miR-365a-3p	0.092	0.39	hsa-miR-342-3p	4.618	0.2
hsa-miR-190a-5p	0.095	0.31	hsa-miR-363-3p	4.526	0.39
hsa-miR-450b-5p	0.105	0.31	hsa-miR-155-5p	4.361	0.27
hsa-miR-125b-5p	0.111	0.23	hsa-miR-130b-3p	3.951	0.27
hsa-miR-499a-5p	0.186	0.12	hsa-miR-193b-3p	3.038	0.3
hsa-miR-532-5p	0.19	0.12	hsa-miR-181b-5p	3.003	0.37
hsa-miR-26a-5p	0.196	0.31	hsa-miR-222-3p	2.749	0.43
hsa-miR-339-5p	0.226	0.36	hsa-miR-132-3p	2.701	0.44
hsa-miR-27a-3p	0.227	0.39	hsa-miR-195-5p	2.653	0.35
hsa-miR-30b-5p	0.242	0.27	hsa-miR-625-5p	2.631	0.19
hsa-miR-188-3p	0.247	0.27	hsa-miR-214-3p	2.488	0.27
hsa-miR-328-3p	0.255	0.23	hsa-miR-181a-5p	2.346	0.33
hsa-miR-29c-3p	0.258	0.27	hsa-miR-598-3p	2.274	0.27
hsa-miR-326	0.258	0.27	hsa-miR-152-3p	2.248	0.35
hsa-let-7b-5p	0.277	0.23			
hsa-miR-32-5p	0.293	0.31			
hsa-miR-140-3p	0.304	0.46			
hsa-miR-660-5p	0.319	0.34			
hsa-miR-194-5p	0.34	0.39			
hsa-miR-502-5p	0.36	0.19			
hsa-miR-30e-5p	0.376	0.39			
hsa-miR-421	0.381	0.48			
hsa-miR-361-5p	0.393	0.36			
hsa-miR-197-3p	0.4	0.34			
hsa-miR-339-3p	0.4	0.31			
hsa-miR-141-3p	0.406	0.24			
hsa-miR-192-5p	0.41	0.19			
hsa-miR-20a-5p	0.467	0.37			

## Data Availability

The datasets generated and/or analyzed during the current study are available from the corresponding authors on reasonable request.

## Supplementary Materials

The following supporting information can be downloaded at: https://www.mdpi.com/article/10.3390/ijms241713556/s1.
