# Supplementary material for: Dynamic Changes in miRNA Expression during the Generation of Expanded and Activated NK Cells
Source: Int J Mol Sci. 2023 Aug 31;24(17):13556. doi: 10.3390/ijms241713556 (PMC10488243; doi:10.3390/ijms241713556)
Supplement: Supplementary file 1 [file ijms-24-13556-s001.zip › ijms-2569427-supplementary.pdf]

Supplementary Material

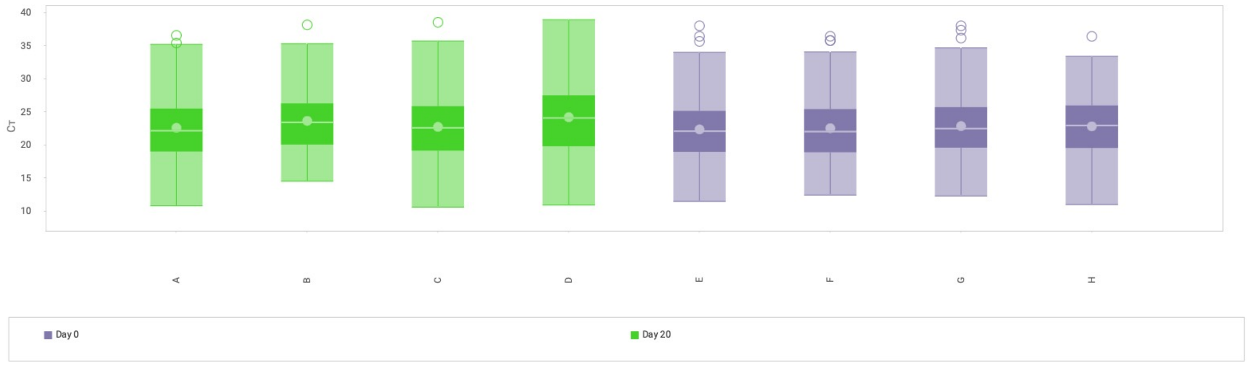

Supplementary Figure S1. CT values for each sample during RT-PCR. All CT values above 35 were not used when calculating ΔΔCTs as they represent miRNAs with expressions too low to be detected through RT-qPCR Green bars indicate the 4 day 20 samples and purple bars indicate the 4 day 0 samples. Pairs are as follow: A-E, B-F, C-G, D-H (day 20 - day 0).

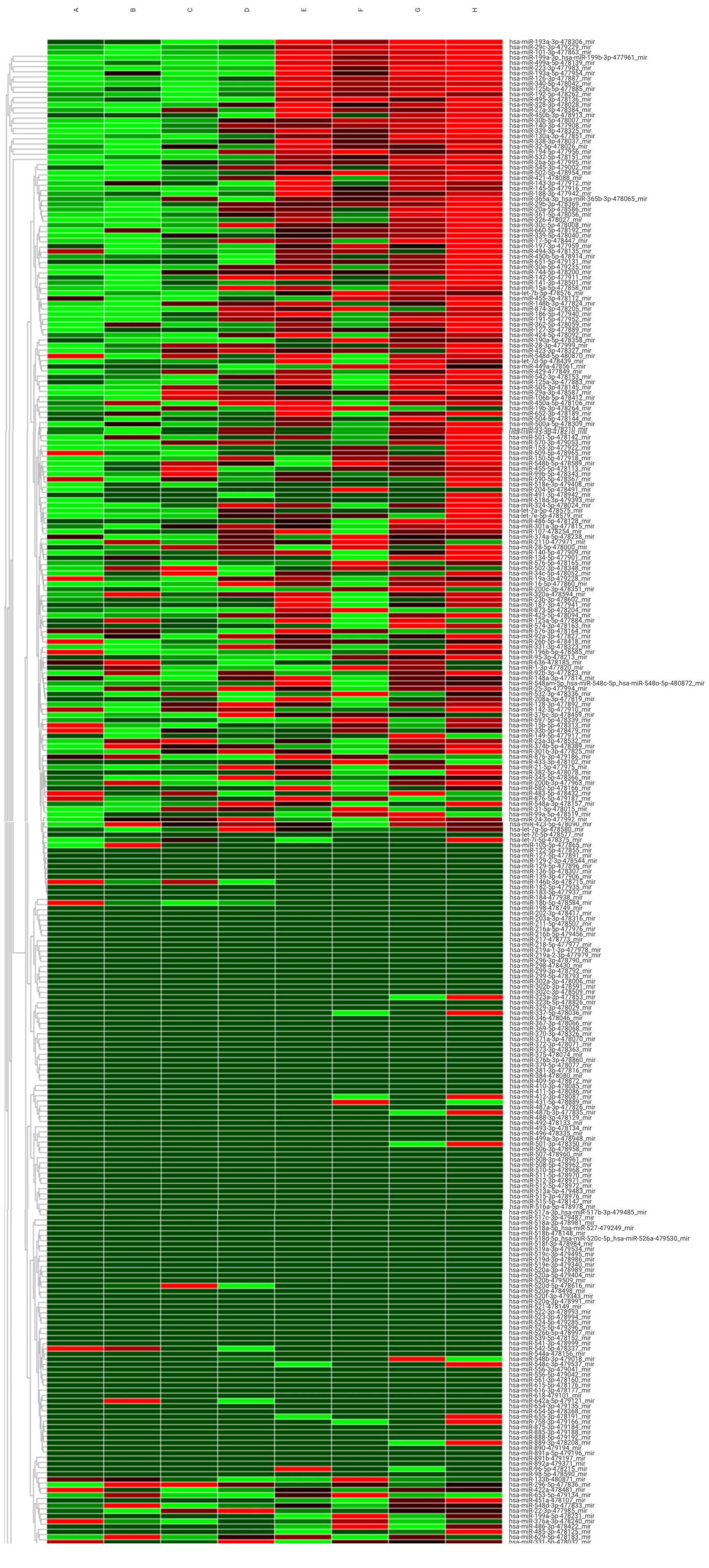
A
B
C
D
E
F
G
H

Supplementary Figure S 2. Heatmaps of the 378 miRNA targets analyzed. Columns represent each sample. A- D are day 20 samples. E-H are day 0 samples. Each row represents a single miRNA target. Red represents an increase in expression and green represents diminishing expression.
